# Reduced risk of diabetic retinopathy in osteoarthritis patients undergoing joint replacement surgery

**DOI:** 10.7150/ijms.109689

**Published:** 2025-04-09

**Authors:** Chin-Te Huang, Yat-Yin Law, Kai Wang, Chia-Yi Lee, Jing-Yang Huang, Shun-Fa Yang, Hsiang-Wen Chien

**Affiliations:** 1 School of Medicine, Chung Shan Medical University, Taichung, Taiwan.; 2 Department of Ophthalmology, Chung Shan Medical University Hospital, Taichung, Taiwan.; 3 Department of Orthopedics, Chung Shan Medical University Hospital, Taichung, Taiwan.; 4 Department of Ophthalmology, Cathay General Hospital, Taipei, Taiwan.; 5 Departments of Ophthalmology, Sijhih Cathay General Hospital, New Taipei City, Taiwan.; 6 School of Medicine, College of Medicine, Fu Jen Catholic University, New Taipei, Taiwan.; 7 Nobel Eye Institute, Taipei, Taiwan.; 8 Department of Medical Research, Chung Shan Medical University Hospital, Taichung, Taiwan.; 9 School of Medicine, National Tsing Hua University, Hsinchu, Taiwan.

**Keywords:** osteoarthritis, epidemiology, database, joint replacement surgery, diabetic retinopathy

## Abstract

Osteoarthritis (OA) is a progressive joint disorder frequently associated with multiple comorbidities. Emerging research suggests a potential link between OA and diabetic retinopathy, a microvascular complication of diabetes mellitus. This study investigates whether joint replacement surgery influences the risk of developing diabetic retinopathy in individuals with OA. Using data from the TriNetX database, we conducted a retrospective cohort study, categorizing OA patients into two groups based on whether they had undergone joint replacement surgery, with each group comprising 164,653 individuals. The primary outcome was the incidence of diabetic retinopathy, analyzed using Cox proportional hazards regression. Among patients who underwent joint replacement surgery, 844 developed diabetic retinopathy, compared to 1,336 cases in the non-surgery group. The incidence of diabetic retinopathy was significantly lower in the surgery group (P < 0.001). Additionally, cumulative incidence analysis confirmed a reduced risk in the surgery group (P < 0.001). Subgroup analyses further demonstrated a consistently lower risk across most demographic subgroups. In conclusion, our findings suggest that joint replacement surgery in OA patients is associated with a reduced risk of developing diabetic retinopathy. Further research is warranted to explore the underlying mechanisms and potential clinical implications.

## Introduction

Osteoarthritis (OA) is a degenerative joint disease that predominantly affects the knees and hips 1. Clinically, OA is characterized by joint pain, transient morning stiffness, and physical disability 2, 3. In severe cases, OA significantly restricts mobility, often necessitating total knee arthroplasty (TKA) or other joint replacement procedures 4, 5. Although surgical outcomes are generally favorable, postoperative pain and the need for analgesic use can influence overall health and recovery 6-8.

Previous studies have established associations between OA and various comorbidities 9. Notably, diabetes mellitus has been linked to an increased incidence of OA compared to non-diabetic populations 10, 11. Additionally, OA is correlated with a higher prevalence of cardiovascular disease and an elevated risk of premature mortality 12. Obesity, particularly in individuals with knee and hip OA, is a well-documented risk factor for disease progression and related complications 13. Moreover, beyond the direct impact of OA, patients undergoing TKA or hip replacement surgery may have an increased risk of postoperative cardiovascular events 14.

Diabetes mellitus affects the vascular system throughout the body, and its microvascular complications include diabetic retinopathy 15-18. Previous research has demonstrated that reducing serum glucose concentrations can lower the incidence of diabetic microvascular complications 19. However, the potential impact of joint replacement surgery on the incidence of diabetic retinopathy in OA patients remains unexplored. Given that joint replacement surgery has been associated with improved life expectancy in OA patients 20, it is plausible that surgical intervention may also influence the progression of diabetic retinopathy, necessitating further investigation.

Therefore, the objective of the present study is to evaluate the incidence of diabetic retinopathy among OA patients who undergo joint replacement surgery compared to those who do not. Additionally, this study aims to assess the correlation between joint replacement surgery and diabetic retinopathy across different patient subgroups.

## Materials and Methods

### Data source

This study adheres to the principles outlined in the Declaration of Helsinki (1964) and its subsequent amendments. Furthermore, it has been approved by the Institutional Review Board of Chung Shan Medical University Hospital (Project Code: CS2-23208). The TriNetX database consolidates de-identified claims data from multiple health insurance providers across the United States, encompassing a diverse population with an estimated coverage exceeding 200 million individuals. This database includes a comprehensive range of medical records, such as International Classification of Diseases, Tenth Revision, Clinical Modification (ICD-10-CM) codes; demographic information (including age, sex, employment status, and geographic region); socioeconomic data; details of medical services received; hospitalization duration; imaging and laboratory test codes; laboratory results; surgical and procedural codes; and Anatomical Therapeutic Chemical (ATC) classification codes for medications.

### Subject selection

**Figure 1:**
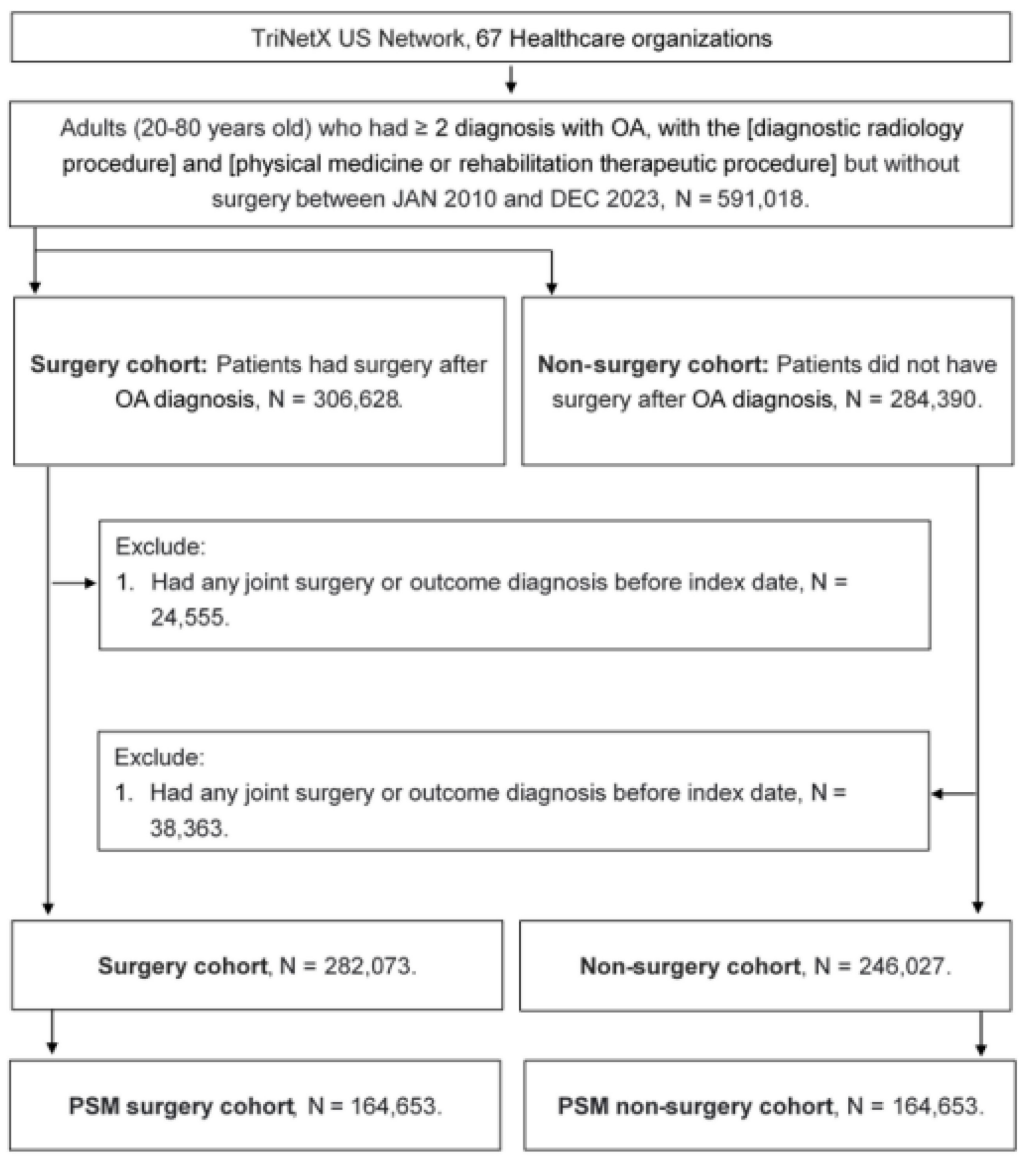
The flowchart of participant selection. N: number, OA: osteoarthritis, PSM: propensity score-matching.

A retrospective cohort study was conducted, and patients meeting the following criteria were identified as having osteoarthritis (OA): (1) age below 20 or above 80 years, (2) receipt of an OA diagnosis based on the corresponding ICD-10-CM codes, (3) completion of a complete blood cell count, white blood cell differential count, and X-ray examination prior to the OA diagnosis, and (4) OA diagnosis confirmed by an orthopedist. The index date was defined as six months after the initial OA diagnosis. To enhance the homogeneity of the study population, the following exclusion criteria were applied: (1) prior history of joint surgery before the index date and (2) occurrence of the study outcome (as defined in the subsequent section) before the index date. Next, patients with OA who underwent joint replacement surgery were matched to OA patients who did not receive joint replacement surgery using the propensity score matching (PSM) method. The PSM approach accounts for demographic characteristics, systemic disorders, and medication history to generate a propensity score, ensuring comparability between matched individuals. Ultimately, 164,653 patients were included in both the surgery and non-surgery groups. The subject selection process is illustrated in Figure 1.

### Main outcome

The primary outcome of this study was the development of diabetic retinopathy. Diabetic retinopathy was defined based on the following criteria: (1) a documented diagnosis of diabetic retinopathy according to the corresponding ICD-10-CM codes and (2) confirmation of the diagnosis by an ophthalmologist. Only diabetic retinopathy events recorded after the index date were considered for outcome assessment. Patients were followed until the occurrence of the outcome, withdrawal from the available health insurance program, or the end of data availability in the TriNetX database (December 31, 2022).

### Confounder adjustment

To comprehensively assess the association between OA-related surgery and subsequent diabetic retinopathy, the following potential confounders were included in multivariable analyses: age, sex, ethnicity, medical encounter frequency, hypertension, nicotine dependence, cerebrovascular disease, alcohol-related disorders, hyperlipidemia, ischemic heart disease, peripheral vascular disease, cerebrovascular disease, chronic lower respiratory disease, body mass index (BMI), estimated glomerular filtration rate (eGFR), serum leukocyte count, serum cholesterol levels, high-density lipoprotein (HDL), low-density lipoprotein (LDL), and glycated hemoglobin (HbA1c). To ensure that systemic disorders had a sufficiently prolonged duration to influence the incidence of diabetic retinopathy, only conditions persisting for more than two years were included in the multivariable analysis.

### Statistical analysis

Statistical analyses were performed using SAS version 9.4 (SAS Institute Inc., Cary, NC, USA). Baseline characteristics were summarized using descriptive statistics, and standardized mean differences (SMD > 0.1) were used to identify significant differences between groups. Cox proportional hazards regression models were employed to estimate the incidence of diabetic retinopathy, with adjusted hazard ratios (aHRs) and 95% confidence intervals (CIs) calculated to account for potential confounders, including demographics, systemic conditions, substance dependence, and laboratory findings. A Kaplan-Meier curve was used to illustrate cumulative incidence, and group comparisons were conducted using the log-rank test. Subgroup analyses were performed by stratifying patients based on age, race, sex, eGFR, and HbA1c, followed by Cox regression to assess subgroup-specific outcomes. Statistical significance was set at P < 0.05, with P < 0.001 considered highly significant.

## Results

The baseline characteristics of the surgery and non-surgery groups are presented in Table 1. The mean age was 64.8 ± 10.5 years in the surgery group and 64.7 ± 11.9 years in the non-surgery group, with no significant difference between the groups (SMD = 0.0019). Similarly, the distributions of sex and ethnicity did not differ significantly between the two groups (both SMD < 0.1). Additionally, the prevalence of systemic comorbidities and substance dependence was statistically comparable between the surgery and non-surgery groups (all SMD < 0.1). Regarding laboratory data, the values were well balanced between the two groups due to the application of the PSM approach (all SMD < 0.1) (Table 1).

**Figure 2:**
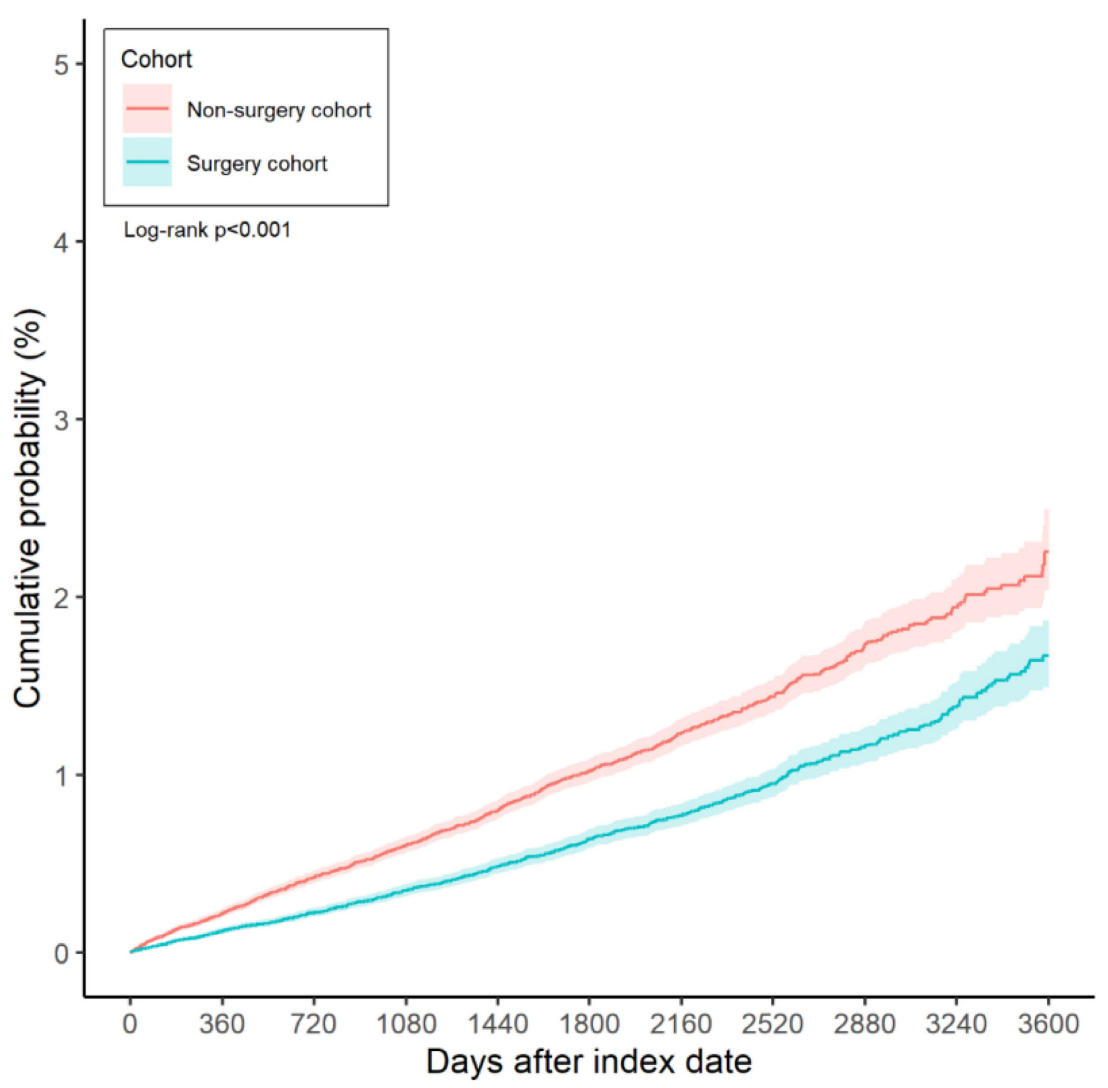
The Kaplan-Meier curve and cumulative incidence of diabetic retinopathy between the surgery and non-surgery groups.

Over the entire study period, 844 cases of diabetic retinopathy were recorded in the surgery group, compared to 1,336 cases in the non-surgery group. According to the results of the multivariable analysis, the incidence of diabetic retinopathy was significantly lower in the surgery group than in the non-surgery group (aHR: 0.633, 95% CI: 0.581-0.690, P < 0.001) (Table 2). Furthermore, the cumulative incidence of diabetic retinopathy was also significantly lower in the surgery group (P < 0.001) (Figure 2).

**Figure 3:**
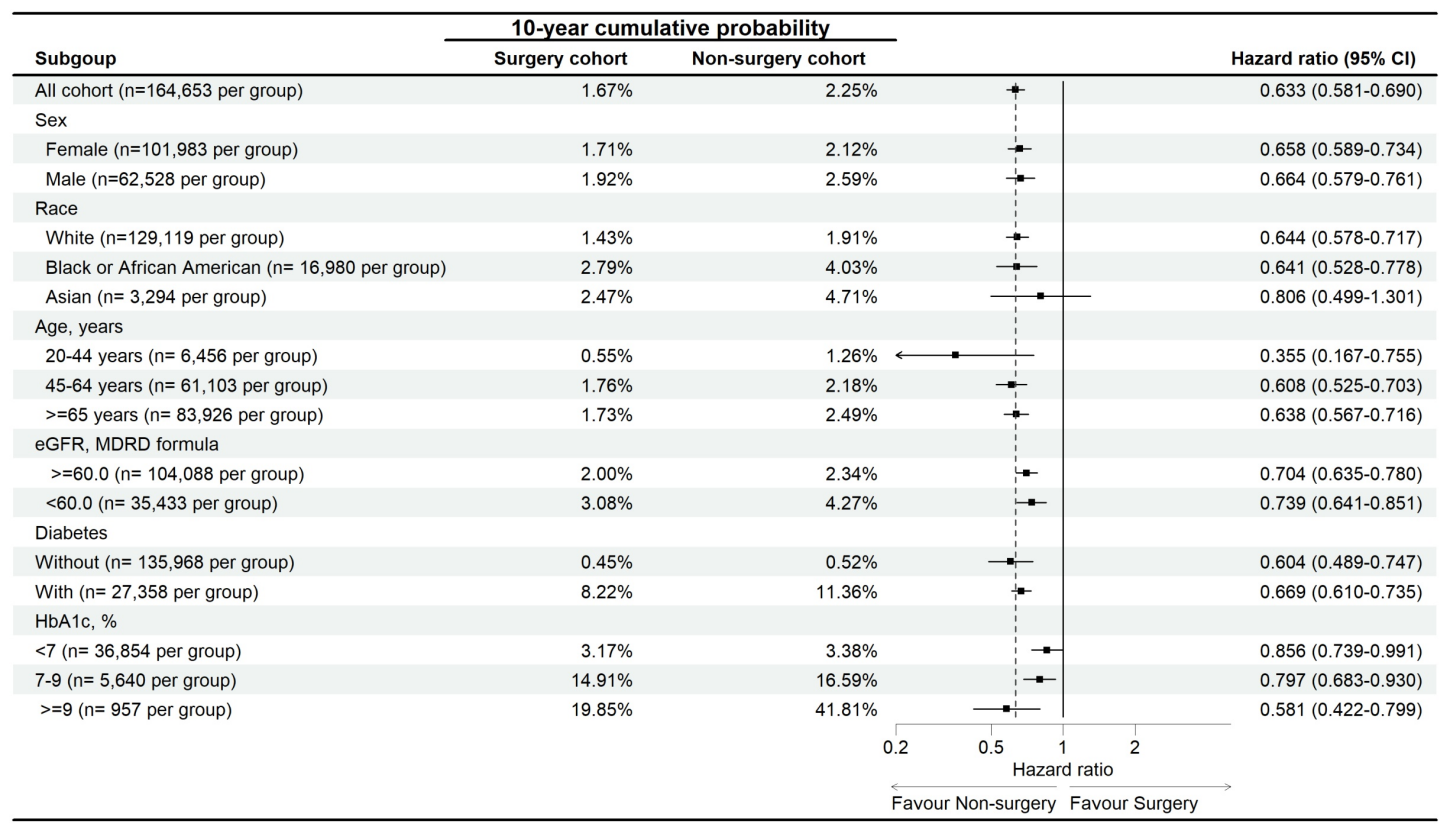
Sensitive analysis of diabetic retinopathy risk in osteoarthritis. eGFR: estimated glomerular filtration rate, HbA1c: glycated hemoglobin, N: number.

In the subgroup analysis, the reduced risk of diabetic retinopathy associated with surgery was observed across most subgroups, except for the Asian population, where the association was not statistically significant (aHR: 0.806, 95% CI: 0.499-1.301) (Figure 3).

## Discussion

The present study demonstrated that the incidence of diabetic retinopathy was significantly lower in OA patients who underwent joint replacement surgery compared to those who did not. Furthermore, the cumulative incidence of diabetic retinopathy was also reduced in the surgery group. Subgroup analysis revealed a consistent protective effect of surgery across different patient characteristics, except in the Asian population, where no significant association was observed.

OA has been linked to multiple pathological mechanisms and comorbidities, as reported in previous literature 9, 10, 21, 22. Mechanical stress and subsequent inflammation play central roles in OA development, with low-grade chronic inflammation persisting in affected joint spaces 23, 24. Recurrent inflammation and neutrophil aggregation contribute to joint damage and OA progression 25, 26. Additionally, individuals with OA exhibit elevated levels of inflammatory biomarkers, such as interleukins and prostaglandins 21, 27. Apart from inflammation, OA pathophysiology involves increased subchondral bone remodeling, oxidative stress, and osteophyte formation 23, 28. Beyond its pathological mechanisms, OA is associated with metabolic disorders, including glucose intolerance and diabetes mellitus 9, 29. Notably, the therapeutic outcomes of TKA in OA patients are significantly influenced by the presence of diabetes mellitus 11.

Moreover, hyperlipidemia is more prevalent in individuals with OA, and the use of lipid-lowering agents has been linked to reduced structural damage in OA patients 30. OA is also correlated with ischemic heart disease, both as a risk factor for its development and as a condition that worsens cardiovascular symptoms 31, 32. Diabetic microvascular complications, including diabetic retinopathy, are characterized by heightened inflammatory responses 33. Patients with diabetic microvascular complications exhibit elevated inflammatory cytokines, such as C-reactive protein 34. Given that both OA and diabetic microvascular complications share an inflammatory etiology and that joint replacement surgery primarily induces localized inflammation 24, 33, 35, we hypothesize that OA patients undergoing surgery may experience reduced systemic inflammation, thereby lowering their risk of developing diabetic retinopathy. This hypothesis is supported by the findings of the present study.

Our results align with previous research indicating that OA patients undergoing joint replacement surgery experience improved long-term health outcomes. A prior study reported that TKA in knee OA patients was associated with a lower mortality rate compared to the general population over a 10-year follow-up period 36. Additionally, joint replacement surgery has been linked to a reduced risk of cardiovascular diseases in OA patients 20. However, limited research has specifically examined the impact of joint replacement surgery on the development of diabetic retinopathy. To our knowledge, this study provides preliminary evidence suggesting a negative correlation between joint replacement surgery and the incidence of diabetic retinopathy in OA patients. To ensure the temporal sequence between surgery and diabetic retinopathy, we excluded participants with a prior diagnosis of diabetic retinopathy before the index date. Additionally, we accounted for key confounding factors related to diabetes mellitus, including age, sex, hypertension, and hyperlipidemia, in the Cox proportional hazards regression model. As a result, joint replacement surgery may serve as an independent protective factor against diabetic retinopathy in OA patients. Beyond its anti-inflammatory effects, surgery may also facilitate increased physical activity by restoring joint function, thereby improving glycemic control, and reducing the risk of diabetic microvascular complications 19, 37. The lower cumulative probability of diabetic retinopathy in the surgery group further supports the notion that persistent OA-related inflammation should be managed to mitigate the risk of diabetic complications.

In the subgroup analysis, joint replacement surgery was associated with a lower risk of diabetic microvascular complications across most patient subgroups, except in the Asian population. Previous studies have shown that TKA reduces the risk of cerebrovascular disease in patients with various characteristics 38. These findings, in combination with the present study, further highlight the potential benefits of joint replacement surgery in reducing comorbidities in OA patients. However, there is limited research addressing the similar incidence of diabetic retinopathy between Asian OA patients with and without surgery. Prior studies suggest that Asian ethnicity is neither a strong predisposing nor a protective factor for diabetic retinopathy 16. A possible explanation for our findings is the small sample size of the Asian population in the TriNetX database, which may have led to statistical bias. Asian participants accounted for approximately 2% of the study population, with a total of only around 3,000 cases--substantially fewer than the African American and Caucasian subgroups. Furthermore, the number of diabetic retinopathy cases in the Asian population was relatively low, with only about 100 recorded cases. The limited sample size may have affected the statistical power of the analysis. Additionally, laboratory data did not significantly influence the incidence of diabetic retinopathy, suggesting that joint replacement surgery may exert a stronger protective effect than other predisposing factors for diabetic retinopathy 16.

OA is a prevalent condition worldwide, with an estimated 300 million cases of hip and knee OA reported in previous studies 39. Radiographic knee OA affects approximately 19% of adults 13, and OA, particularly knee OA, is a leading cause of disability globally 40. Among patients with end-stage OA, joint replacement surgery is a commonly recommended intervention, albeit associated with substantial healthcare costs 41. In the United Kingdom alone, over 60,000 TKA procedures are performed annually 6. Similarly, diabetes mellitus is a widespread disease affecting approximately 500 million individuals worldwide 15. Furthermore, diabetic microvascular complications impact more than 20% of individuals with diabetes mellitus 16, contributing to severe outcomes such as blindness and end-stage renal disease requiring dialysis--both of which impose significant socioeconomic burdens 19. Given the substantial health impact of both OA and diabetic retinopathy, identifying strategies to reduce the risk of diabetic retinopathy in OA patients is of clinical and public health importance.

Despite its strengths, this study has several limitations. First, the TriNetX database is a claims-based dataset, meaning that only coded diagnoses are accessible, rather than complete medical records. Consequently, critical clinical details--such as radiographic findings, OA severity, affected joint sites, surgical techniques, postoperative alignment, range of motion, diabetic retinopathy severity, treatment response, and recurrence--could not be assessed. Second, the retrospective design may introduce heterogeneity in patient health status and disease severity, despite the application of PSM. Additionally, inflammatory biomarkers were not available in the dataset, limiting our ability to confirm the exact mechanisms underlying the reduced incidence of diabetic microvascular complications in the surgery group.

In conclusion, joint replacement surgery is associated with a lower risk of developing diabetic retinopathy in OA patients. Furthermore, the incidence of diabetic retinopathy is inversely correlated with the duration of OA in patients undergoing surgery compared to those who do not receive surgery. These findings suggest that early joint replacement surgery may be beneficial for OA patients with diabetes mellitus. Future large-scale prospective studies are warranted to further explore the relationship between joint replacement surgery and diabetic retinopathy outcomes in OA patients.

## Figures and Tables

**Table 1:** Baseline characteristics between the two cohorts after propensity score matching

Characteristics	Surgery cohort	Non-Surgery cohort	SMD
N	164,653	164,653	
Age at Index	64.8±10.5	64.7±11.9	0.0019
Sex			
Female	101362(61.6%)	101626(61.7%)	0.0033
Male	63274(38.4%)	63013(38.3%)	0.0033
Race			
Caucasian	128630(78.1%)	128266(77.9%)	0.0053
African	17103(10.4%)	17667(10.7%)	0.0111
Asian	3883(2.4%)	3740(2.3%)	0.0058
Medical encounter			
Preventive medicine services	11540(7.0%)	11536(7.0%)	0.0001
Inpatient encounter	87083(52.9%)	45424(27.6%)	0.5340
Emergency	24386(14.8%)	26032(15.8%)	0.0278
Critical care services	1643(1.0%)	7620(4.6%)	0.2209
Comorbidities			
Nicotine dependence	14423(8.8%)	14657(8.9%)	0.0050
Alcohol related disorders	3882(2.4%)	4069(2.5%)	0.0074
Hypertensive diseases	91357(55.5%)	91137(55.4%)	0.0027
Hyperlipidemia	72534(44.1%)	71950(43.7%)	0.0071
Ischemic heart diseases	21198(12.9%)	21253(12.9%)	0.0010
Peripheral vascular disorders	12316(7.5%)	12718(7.7%)	0.0092
Chronic lower respiratory diseases	28377(17.2%)	28685(17.4%)	0.0049
Cerebrovascular diseases	7651(4.6%)	8176(5.0%)	0.0149
Lab data			
BMI	30.7±6.4	30.9±7.8	0.0369
eGFR	77.9±21.9	78.2±25.5	0.0121
Serum leukocytes	7.5±19.7	8.6±37.9	0.0336
Serum cholesterol	181.6±45.9	178.6±49.5	0.0646
HDL	53.9±19.8	52.1±19.1	0.0911
LDL	101.9±37.1	101.1±38.7	0.0199
HbA1c	5.9±0.9	6.1±1.4	0.1932

BMI: body mass index, eGFR: estimated glomerular filtration rate, HbA1c: glycated hemoglobin, N: number, SMD: standard mean difference.

**Table 2:** Main outcomes between the surgery and non-surgery groups

Study event	N	Cumulative probability	Cumulative probability	Cumulative probability	Cumulative probability	aHR (95% CI)	P
Study event	N	1-year	3-year	5-years	10-years	aHR (95% CI)	P
Diabetic retinopathy							
Non-surgery cohort	1,336	0.22%	0.60%	1.02%	2.25%	Reference	
Surgery cohort	844	0.12%	0.35%	0.64%	1.67%	0.633(0.581-0.690)	<0.001*

aHR: adjusted hazard ratio, CI: confidence interval, N: number. * denotes significant difference between groups.
